# Population Immunity and Polio Eradication

**DOI:** 10.3390/pathogens13030183

**Published:** 2024-02-20

**Authors:** Paul E. M. Fine

**Affiliations:** Department of Infectious Disease Epidemiology and International Health, London School of Hygiene and Tropical Medicine, Keppel St., London WC1E 7HT, UK; paul.fine@lshtm.ac.uk

**Keywords:** Polio, eradication, immunity, herd immunity, vaccination

## Abstract

The Global Polio Eradication Initiative made immense progress after its establishment in 1988 as a consequence of high coverage with various poliovirus vaccines in all populations of the world. Problems have arisen in recent years, however, related to security issues in some countries, to the circulation of vaccine-derived polioviruses, and to the recognition that individuals with certain immune deficiencies can remain infected and infectious for many months or years. As natural infection and different vaccines have different effects on the immune system, the patterns of humoral and mucosal immunity to polioviruses in the world today are complex but are crucial to the ultimate success of the eradication initiative. This paper describes the background of the current situation and current immunological patterns and discusses their implications for managing population immunity to polioviruses in the years ahead.

## 1. Introduction

The global polio eradication initiative was established as a millennium target by the World Health Assembly in 1988. Its justification was based upon a mixture of theory and experience. The theory claimed that, by raising immunity above a defined “herd immunity threshold” level, infection incidence should decline—ultimately to zero, if that high immunity level could be maintained. The experience had been provided by the successful eradication of smallpox, as achieved and certified in the late 1970s. In addition, the Pan American Region of the WHO was making impressive progress toward a regional polio elimination target, set in 1985, after discovering the effectiveness of vaccination campaigns. The argument for the feasibility of eradication was bolstered by the presence of the Expanded Programme on Immunisation (EPI), which was providing routine immunisation services to all populations. The argument was also supported by economic models, which showed that the additional costs of a targeted programme could be more than recovered by future savings if polioviruses ceased to circulate and vaccinations could be discontinued worldwide [1,2].

As documented in the several papers in this issue, there was dramatic progress towards the eradication goal for 12 years after it was set. The incidence of poliomyelitis disease declined globally by approximately 99% by the year 2000. Types 2 and 3 wild virus stopped circulating in 1999 and 2012, respectively, and their eradication has been certified. However, major difficulties have appeared: political problems disrupting vaccination programmes in several countries, the appearance and extensive spread of vaccine-derived polioviruses, now circulating in some 20 countries, and the recognition of the long-term secretion of polioviruses by individuals with certain immune deficiencies. These observations have challenged the theoretical and practical arguments that had been used to support the eradication initiative.

The declines in infection and disease incidence have been brought about entirely by vaccination, on a massive scale, in every population in the world. The nature and extent of the immunity induced by this vaccination initiative are complex but critical for the ultimate success or failure of the entire initiative. This paper summarises the theoretical and practical background to the evolving circumstances, mentions some outstanding gaps in our understanding of population immunity to polioviruses, and considers their implications.

## 2. Theoretical Background

There is a large library of literature devoted to the theory of infection eradication by means of vaccines [3]. Much of this literature emphasises the concept of “herd immunity”, defined as a threshold level of immunity in a population above which infection incidence should decline, ultimately (if levels above that threshold are maintained) to zero.

The basic argument is simple. It starts with a recognition that the level of immunity required in a population to reduce or stop transmission of an infectious agent is related to the transmissibility of that infection: the more transmissible it is, then the higher the level of immunity required. Transmissibility in this context is most appropriately described by the “basic reproduction number”, typically symbolised as *R*_0_, and defined as the average number of transmissions by each infected individual in a totally susceptible population. In numerical terms, if each infected individual would transmit the agent on average to *R*_0_ others in a totally susceptible population, then infection incidences would be constant if all but one of the *R*_0_ (i.e., [*R*_0_ − 1] out of the *R*_0_) individuals exposed to each infectious individual were immune and protected against infection. This gives a threshold for the critical proportion immune, often called the herd immunity threshold:

(*R*_0_ − 1)/*R*_0_ = 1 − 1/*R*_0_ (1)

**Figure 1:**
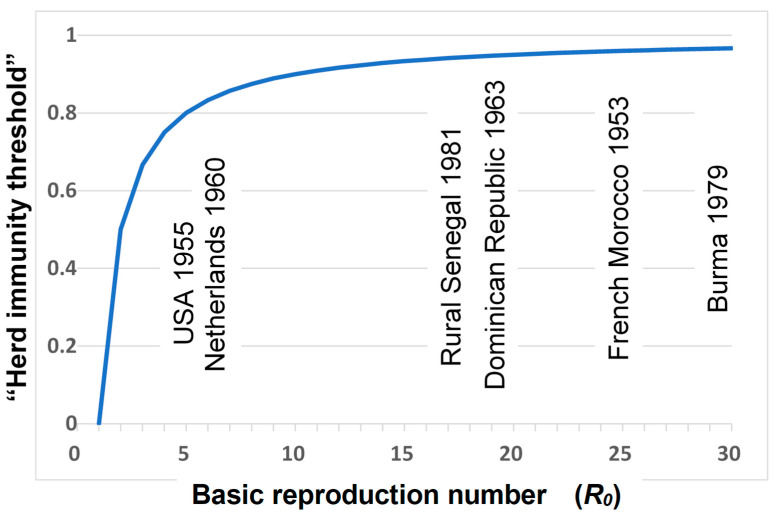
Estimates of the basic reproduction numbers and simple theoretical “herd immunity thresholds” (calculated as: 1 − 1/*R*_0_) for wild poliovirus, in different populations, from References [4,5].

This relationship is illustrated in Figure 1. The implication of such a threshold is that, as long as the proportion immune is above this threshold, then incidences of infection should decline in the population, ultimately to zero.

Because of this relationship, there has been much interest in estimating the *R*_0_ of different infections, including polioviruses. These estimations were based upon the age patterns of infection and immunity in populations in which the infection was at a stable endemic level (as constant incidences imply that the proportion immune in the population is at the herd immunity threshold). Starting with the simplest possible assumption, if everyone became infected (and, thus, naturally immune) at age A and lived for L years, then A/L would give the proportion of everyone’s life spent susceptible and 1 − A/L would be the proportion immune. Equating this to the herd immunity threshold (expression 1) gives A/L = 1/*R*_0_ and thus *R*_0_ = L/A. In other words, the basic reproduction number should be equal to the average life expectancy divided by the average age at infection. It was later shown by Dietz that this estimate can be improved by assuming that mortality is constant by age rather than assuming everyone lives for L years; in which case, the basic reproduction number should be *R*_0_ = L/A + 1 [3,6].

The application of this theory to poliovirus serological data, from studies carried out before vaccines were introduced, provided estimates of *R*_0_ ranging from approximately 4 for wealthy countries up to 30 for poor populations (see Figure 1). One should not expect precision in such estimates, but this range of values makes intuitive sense, given the faecal–oral mode of transmission of polioviruses, which means that transmission is far more efficient (*R*_0_ is far higher) in poor populations with inadequate standards of hygiene than in wealthy populations with high standards of sewage disposal and hygiene practices [4,5,7].

On the basis of such logic, it was estimated that the herd immunity threshold for poliovirus eradication ranges from approximately 75% in wealthy high-hygiene populations to 97% in poor populations [4,5,6]. The theoretical challenge of the eradication programme was thus to achieve, and maintain, these levels of immunity with vaccines.

Such simple logic appears in many textbooks. It may be appealing, but reflection reveals a variety of problems in terms of its applicability to actual circumstances. It implies several crucial assumptions.

1. There is an assumption that immunity is 100% effective in preventing infection and infectiousness. However, immunity versus poliovirus infection is complicated, depending upon whether it is attributable to natural infection, inactivated vaccines or oral vaccines, or to combinations of these over time [5,8]. It is well known that immunity induced by oral poliovirus vaccine (OPV) is qualitatively very different from that induced by inactivated poliovirus vaccine (IPV) and that OPV induces far stronger intestinal mucosal IgA-mediated protection than IPV [9]. Thus, while both types of vaccines may be highly effective in preventing disease, OPV is far more effective in preventing infection and infectiousness, which are essential for stopping their transmission and, hence, eradication. This is a major reason why most countries relied upon OPV in their eradication programmes, the only exceptions being a few countries (e.g., the Netherlands) with very high standards of hygiene and hence very low *R_o_* for polioviruses. The distinction was illustrated dramatically by circumstances in Israel, which shifted from OPV to IPV in 2005, and where a wild virus circulated for several months in the absence of the clinical disease in 2013, necessitating the national programme to revert to the use of OPV vaccines [10].

Though OPV-derived intestinal mucosal immunity is far stronger than that generated by IPV, challenge studies have shown that it is not complete or 100% protective, i.e., OPV-vaccinated individuals still become infected when challenged and still excrete small numbers of polioviruses. Interestingly, it may be that enhanced-potency IPV vaccines are more effective in providing pharyngeal rather than intestinal mucosal immunity [11]. There is also heterogeneity in vaccine responses between populations. Unfortunately, the responses to OPV are weaker in low-income tropical populations than in high-income temperate zone areas, perhaps because of interference from other enteric infections. This means they perform least well in those areas with the highest poliovirus transmissibility [5].

A further complexity arises with reference to poliovirus immunity in that there is some level of cross-protection between types. This is a complex issue that has proved difficult to study but might have to be considered in a thorough assessment of population immunity.

2. There is an assumption in discussions of herd immunity and eradication that immunity persists and does not wane. The duration of protection induced by IPV vaccines is still under discussion, but several high-income countries recommend boosters for adults [8]. Studies in India have revealed that mucosal immunity declines appreciably with time after OPV [12]. The recognition of the long-term excretion of polioviruses by individuals with particular immune deficiencies means that the duration of protection is a crucially important issue for the eradication initiative [13].

3. There is also an assumption in basic herd immunity calculations that populations are homogeneous and that they mix at random. This is, of course, far from true in real populations. People and communities are heterogeneous, and they do not mix at random. There are subsets in all populations made up of individuals who mix more intensely than others in terms of the sorts of contact required for the transmission of polioviruses. These characteristics are typically associated with socioeconomic conditions and mean that certain subsets of any large population can continue to transmit and maintain an infection to which many others in the larger population are effectively no longer at risk. This circumstance is exacerbated in many populations by the fact that vaccination coverage levels differ between different groups, and it is often the fact that relatively poor and underserved segments of populations have relatively low levels of vaccination coverage, as well as relatively low levels of sanitation and hygiene. The growth of vaccine hesitancy sentiment in many populations adds a further complexity to this issue—illustrated, for example, by the recently documented circulation of vaccine-derived viruses—and a clinical case in a population in New York with low level of vaccine uptake [14]. The fact that the large majority of wild polio virus activity in Pakistan has been concentrated in the Pashto ethnic group, who constitute only some 15% of the national population, is a particularly important example of this heterogeneity today [15].

For all these reasons, simplistic estimations of herd immunity thresholds are misleading and typically underestimate the actual levels of immunity required to continually reduce the incidence of infection in populations. It is possible to make “improved” theoretical estimates based upon the incorporation of a range of assumptions relating to the quality of immune response and non-random distribution of risk and of vaccines into the equation, but the variety of potential and actual circumstances is large and difficult to define with any confidence. Given the complexity of the different vaccines and schedules in the past and current use and the variety of immunological measures, it is very difficult to define the level of immunity in any population. No single measure would suffice.

Many studies have provided measures of IgG seroprevalence in different populations [16,17]. While useful as indicators of previous exposure to natural infection and to vaccines, these seroprevalence levels are difficult to interpret for two reasons. First, they do not measure mucosal immunity, which is crucial for protection against infection transmission. Secondly, these levels typically differ by age and social group, but the roles of these different groups in maintaining infection transmission relate to their hygiene and other risk behaviour and are unclear.

Notwithstanding such criticisms, the basic principle of herd immunity has proved valid in the context of the polio eradication programme, as demonstrated by the dramatic declines in poliovirus circulation achieved in many populations around the world and the fact that several high-income countries have been free of poliovirus circulation for many years. But the simple herd immunity theory is no longer helpful with the challenge facing the polio eradication initiative today. The real focus of the eradication programme needs to be on achieving and maintaining high routine immunisation coverage against polio in all populations and high coverage during supplemental immunisation activities (SIAs) and assuring high-quality surveillance to detect the virus when it is circulating so that rapid action can be taken to terminate transmission.

## 3. Practical Realities—Current

The current vaccination policies do not aim to achieve some specified prevalence level of some particular measure of population immunity. There has been little discussion of numerical herd immunity thresholds in recent years. It is now more appropriate to discuss different aims and challenges of polio vaccination dependent upon national or regional circumstances. This may be considered in terms of three broad sets of circumstances, analogous to the “goals” as described in the current WHO Strategic Plan 2022–2026 [18].

### 3.1. Populations with Continued Transmission of the Wild Virus (i.e., Afghanistan and Pakistan Today)

The manifest difficulty in stopping the last chains of wild poliovirus type 1 (WPV1) transmission in these countries reflects a particularly complex interaction of social diversity (for example, the concentration of wild virus transmission in the Pashto population), epidemiological risk factors associated with contact and hygiene (and thus high *R*_0_), political insecurity, and an extreme resistance to vaccination in certain population groups. The national programmes have worked hard to carry out both acute flaccid paralysis (AFP) and environmental surveillance in their populations and to raise coverage as high as possible using the bivalent 1–3 OPV vaccine—in particular, in those communities where wild virus is found. This vaccine is used because it is available, though the type 3 component is no longer needed in this context. Though the challenge of stopping WPV1 circulation has stymied these national programmes for several years, there is now some hope of success in that case numbers are lower in 2023 than ever before, and we may hope that the control efforts will at last succeed. Once the WPV1 virus is no longer found, there will be a need to maintain a high level of OPV-derived immunity for at least three years of intensive surveillance until WPV1 eradication is certified. There are arguments to shift to type 1 novel oral poliovirus vaccine (nOPV1) (and perhaps, also, nOPV3 vaccines), when available, to minimise the risk of generating type 1 or 3 vaccine-derived polio viruses (VDPVs).

### 3.2. Populations with Circulating VDPVs

This applies now (late 2023) to some 23 countries, 4 with circulating cVDPV1 and 20 with cVDPV2. The incidence of cVDPV2 increased dramatically, especially in Africa, after the removal of Sabin type 2 from trivalent OPV in 2016. The response to these events was initially to carry out monovalent OPV2 campaigns, but the availability of nOPV2 vaccines has provided a better alternative, with appreciably less risk of generating further VDPVs [19]. More than 800 million doses of nOPV2 have been used in 16 countries by late 2023, reducing the number of (reported) cVDPV2 cases from 689 in 2022 to 364 in 2023. Whether nOPV2 use will be sufficient to stop all cVDPV2 circulation is not yet known, as there is some evidence that nOPV2 can itself produce new vaccine-derived strains [20]. There has been a recent substantial increase in cVDPV1, which increases the need for regulatory approval of the nOPV1 vaccine with a low tendency to revert towards wild-type virulence.

### 3.3. Populations with No Circulating Polioviruses

The majority of countries are now in this category. All continue routine poliovirus vaccination, with a variety of vaccines and schedules [5]. Most low- and middle-income countries (LMICs) have a schedule involving three doses of bivalent 1–3 OPV, starting at a minimum of 6 weeks, supplemented with a dose of trivalent IPV administered from a minimum of 14 weeks of age (with DTP3 or Penta3). High-income counties use only trivalent IPV, typically combined with other antigens (e.g., as a hexavalent product with diphtheria, tetanus, pertussis, *Haemophilus*, and hepatitis B antigens). Several of these countries have found evidence of the circulation of VDPV in recent years by means of environmental surveillance and have responded in a variety of ways. In addition to the New York example mentioned above, type 2 VDPV was identified in sewage in North London in 2022, which led to a response whereby 370,000 children ages 1–9 received an additional dose of either hexavalent or trivalent (with diphtheria and tetanus) IPV vaccine [21].

The overall implication of these activities and history is an immensely complex pattern of immunity to polioviruses in today’s world. Immunity levels are high, but the precise prevalence of particular immunoglobulins and intestinal immunity to particular virus types varies considerably within and between populations.

## 4. Practical Implications—Future

Given the high level of international travel in today’s world, as long as any wild or vaccine-derived poliovirus continues to circulate anywhere, the entire global population will remain at risk of poliovirus infection and polio disease, and it is likely that all countries will consider it necessary to continue vaccination of their populations to maintain a high level of protective “immunity”. The vaccines used will differ between different regions of the world.

It is likely that high-income countries (HICs) will continue to use only IPV, as they are highly effective against disease and have been shown to provide sufficient mucosal immunity to stop poliovirus transmission where standards of hygiene are high. In addition, they avoid the risk of vaccine-associated paralytic polio (VAPP) or of generating VDPVs. These vaccines are relatively expensive, but HICs are relatively risk-averse and able to afford them. The recent experiences of Israel, the USA, and the UK with the circulation of polioviruses (WPV1 in Israel, cVDPV2 in the USA, and the UK) in populations with IPV-only policies are important indicators here. They demonstrate that IPV can mask continued poliovirus transmission by selective prevention of the disease. IPV can only stop poliovirus transmission if at high coverage in populations with very high standards of hygiene, but even in these circumstances, there may be subpopulations with low coverage and/or particular socioeconomic-related risk factors in which circulation can persist.

In contrast to the HICs, most LMICs are likely to continue with live oral vaccines for the foreseeable future. Perhaps the most important outstanding question is whether the newly developed nOPV vaccines will be able to stop the circulation of the current cVDPV viruses without introducing new circulating VDPVs themselves.

This presents a dilemma, as the continued use of OPV vaccines of any sort in LMICs means that live transmissible viruses continue to be introduced with the attendant risk of generating new cVDPVs. Opinions differ as to the long-term implications of this situation. Chumakov et al. recently argued that one should plan for the continued use of new OPV vaccines with a minimal tendency to create cVDPVs and that the overall goal of the programme should shift from the eradication of infection to the prevention of disease. They note that there is some evidence that oral polio vaccines can have beneficial nonspecific effects and that this should be taken into consideration in setting policies [22,23].

A major determinant of future policies, and of the global epidemiological circumstances, will be determined by the properties and availability of different sorts of vaccines [5,8]. The development and introduction of nOPV2 in recent years has helped to reduce the transmission of type 2 VDPVs. Analogous nOPV1 and nOPV3 vaccines are in development and will hopefully be available in the near future. In addition, there are efforts to develop other sorts of vaccines, including based upon mRNA or vaccine-like particle (VLP) technology. We may hope that one or another of these vaccines proves able to provide mucosal protection but avoid any risk of generating further VDPVs.

Whatever vaccines are available, several complex issues must be taken into consideration in determining poliovirus vaccination policies.

1. The properties of the available poliovirus vaccines. This includes their production properties and cost, as well as type specificity, effectiveness, and safety profile, and the willingness of international agencies such as the Global Alliance for Vaccines and Immunisation (GAVI) to provide them to LMICs.
2. The capabilities of different countries to provide vaccines to their populations with high routine coverage.
3. The need for campaigns or “national immunisation days” (NIDs). These have been a regular feature of polio control for the past 20 years at considerable cost and disruption. Costs for these activities should not fall only on LMICs, where poliomyelitis is not among the highest-priority health concerns.
4. Concerns over the reintroduction of polioviruses in the future. This raises issues of (a) containment of polioviruses in laboratories and vaccine production facilities throughout the world, (b) the presence of long-term and immune deficient virus excretors, and (c) the risk of the de novo synthesis of new polioviruses and bioterrorism
5. The extent and effectiveness of global poliovirus surveillance, both AFP and environmental surveillance. This is a major global undertaking and is expensive.
6. The acceptability of polio vaccines in a world with increasing vaccine hesitancy, recognising that polio vaccines have been the targets of particular distrust in some populations.
7. The nature and credibility of strategies for a stepwise focus upon one or another poliovirus type, e.g., withdrawal of type 3 antigens from OPVs and IPVs. These strategies have major implications for vaccine production and supply.
8. The credibility of evidence that OPV vaccines can have beneficial nonspecific effects on child mortality.
9. The propensity of nOPV vaccines to produce cVDPVs, albeit at a markedly reduced rate.
10. Competing public health issues (COVID-19 has provided a dramatic example).
11. The contribution of polio programme resources to other public health problems. This has long been recognised as necessary for the acceptability of polio eradication activities—in particular, in low-income settings.
12. The need to intensify surveillance, including water and sewage surveillance, to detect any polioviruses, which can lead to appropriate responses to stop transmission before it becomes widespread.
13. The future of special funding for polio at the global level.

Consideration of all these issues together is immensely challenging and made even more difficult by the fact that the debate is held in a world in which many other important health and political issues compete for resources. Polio eradication has held a special place among global health priorities for more than three decades, but this cannot continue forever.

## 5. Discussion

The goal of global polio eradication was set 35 years ago. We may remain committed to having it succeed, but that outcome is by no means certain.

Regardless of the long-term outcome of the initiative, it is important to acknowledge the success in reducing a major cause of disability from some 350,000 cases per year by at least 99% [5]. For that, the world may and should be grateful. But it is also important to learn many lessons from the experience of the initiative: that eradication is far more difficult and complicated to achieve than is expressed in simple herd immunity thresholds.

In this context, it is appropriate to harken back to the smallpox eradication example [24]. It was achieved in just eleven years after the WHO set a ten-year target in 1966 and was thus relatively simple compared to the polio experience, But it is notable that the leaders of that initiative all commented on how lucky they were, given a variety of unforeseen political and financial challenges [25,26].

Though global success with smallpox eradication provided an example for establishing the global polio eradication initiative, the viruses and associated challenges were very different for the two diseases. In particular, the low case-to-infection ratio of polioviruses contrasts with the fact that virtually all variola infections are/were clinically manifest and relatively easy to diagnose. Surveillance was thus vastly simpler for variola than for polioviruses. In addition, a single dose of variola vaccine was sufficient. Further, smallpox virus transmission was predictable. Field workers could identify cases and their contacts with relative ease. They could then focus their immunisation efforts on those contacts. However, the transmissibility of polioviruses is appreciably greater than that of variola in poor populations, and the location of transmission is not predictable, given that the vast majority of transmitters have no symptoms at all. For these reasons, many countries were confident about discontinuing smallpox vaccination some years prior to global eradication. This has not been the case for polio. Few, if any, countries will be willing to stop poliovirus vaccination until after a certification of global eradication, and some may recommend vaccination in their populations for some years thereafter.

There remains a question of the levels of population immunity against polioviruses that will or would be called for in different populations if eradication is not achieved and wild or vaccine-derived viruses continue to circulate. There will be an argument for all countries to maintain very high routine coverage with vaccines similar to those used today (e.g., IPV in HICs and OPV supplemented by IPV in LMICs). In the absence of eradication, there will continue to be outbreaks in high-risk population subsets with low vaccination coverage, requiring responses. These outbreaks may become large, affecting many countries, if high vaccine coverage levels, good surveillance, and a strong response capacity are not maintained. The nature of the responses will be dependent upon the virus involved, the size and nature of the affected group, and the vaccination policy preferred by the population and is likely to mean targeted campaigns with IPV or a type-specific nOPV (or some new sort of vaccine). The coverage requirement will depend on the environmental and social circumstances and vaccination history. Optimising the management of these outbreaks will call upon the accumulated wisdom from experience of poliovirus control in many settings over recent decades.

Should polio eradication not occur in the near future, we may hope for a future world in which socioeconomic conditions and public health infrastructures are improved everywhere and sufficient mucosal immunity is achievable, so that the final eradication of polioviruses can be achieved as readily as in the high- and middle-income countries of today.

## Data Availability

Not applicable.
